# Effect of Frequency–Amplitude Parameter and Aspect Ratio on Propulsion Performance of Underwater Flapping-Foil

**DOI:** 10.3390/biomimetics9060324

**Published:** 2024-05-28

**Authors:** Hao Ding, Ruoqian Chen, Yawei Zhu, Huipeng Shen, Qiang Gao

**Affiliations:** 1 Henan Key Laboratory of Superhard Abrasives and Grinding Equipment, Henan University of Technology, Zhengzhou 450001, China; dinghao@haut.edu.cn (H.D.); hpengshen@haut.edu.cn (H.S.); 2 School of Mechanical and Electrical Engineering, Henan University of Technology, Zhengzhou 450001, China; 15378775937@163.com (R.C.); gaoqiang0985@gmail.com (Q.G.)

**Keywords:** flapping-foil, hydrodynamic, frequency–amplitude motion, aspect ratio, propulsive efficiency

## Abstract

The propulsion system is the core component of unmanned underwater vehicles. The flapping propulsion method of marine animals’ flippers, which allows for flexibility, low noise, and high energy utilization at low speeds, can provide a new perspective for the development of new propulsion technology. In this study, a new experimental flapping propulsion apparatus that can be installed in both directions has been constructed. The guide rail slider mechanism can achieve the retention of force in the direction of movement, thereby decoupling thrust, lift, and torque. Subsequently, the motion parameters of frequency–amplitude related to the thrust and lift of a bionic flapping-foil are scrutinized. A response surface connecting propulsion efficiency and these motion parameters is formulated. The highest efficiency of the flapping-foil propulsion is achieved at a frequency of 2 Hz and an amplitude of 40°. Furthermore, the impact of the installation mode and the aspect ratio of the flapping-foil is examined. The reverse installation of the swing yields a higher thrust than the forward swing. As the chord length remains constant and the span length increases, the propulsive efficiency gradually improves. When the chord length is extended to a certain degree, the propulsion efficiency exhibits a parabolic pattern, increasing initially and then diminishing. This investigation offers a novel perspective for the bionic design within the domain of underwater propulsion. This research provides valuable theoretical guidance for bionic design in the underwater propulsion field.

## 1. Introduction

The unmanned underwater vehicle (UUV), as an underwater unmanned intelligent mobile platform, has broad application prospects and huge potential value in the exploration of marine resources, marine scientific investigations, and in the military and other fields [1]. The technology of UUVs involves many disciplines and fields; one of the key technologies is the underwater propulsion [2,3,4]. Traditional propeller propulsion technology has irreplaceable advantages in practical applications, and the theoretical research and practical applications have been mature. However, due to the defects of large noise, large disturbances to the environment, and poor movement flexibility and concealment, the application of propellers is limited.

With the rapid development of bionics [5,6], fluid mechanics, computers [7], and intelligent control technology, as well as the emergence of new materials, scholars have gradually turned to the research of aquatic animal motion mechanisms to seek new underwater propulsion methods [8,9,10,11,12]. After hundreds of millions of years of evolution, underwater organisms have evolved various capabilities adapted to water movement [13,14,15]. Unlike the widely used biomimetic fin swing propulsion technology [16], bionic flapping-foil propulsion is generally generated by simulating the front limb flutter of large aquatic animals. The bionic flapping-foil and unmanned underwater vehicle developed by imitating the fin limbs of marine organisms such as sea turtles [17] and dolphins [18] and their motion modes have high propulsion efficiency and maneuverability, which can provide a new idea for the development of new propulsion technology.

At present, the research methods of bionic flapping-foil propulsion technology are mainly numerical simulations and experimental research. The numerical simulation method has promoted our understanding of bionic flapping-foil propulsion to a certain extent [19,20,21]. Pedro et al. [22] used the Computational Fluid Dynamics (CFD) method to study two-dimensional flapping propulsion characteristics. They found that the inertia force at a low Reynolds number has a much greater influence on the flapping thrust and lift than the viscous force and pointed out that the parameters such as maximum pitch angle, phase angle, and Strouhal (*St*) number are very sensitive to the flapping thrust and propulsion efficiency. Ashraf et al. [23] explored the influence mechanism of the Reynolds number, flapping-foil thickness, and arch curvature on propulsion performance by comparing the results of the viscous Navier–Stokes method with those of the non-viscous panel method. Tang et al. [24] carried out a numerical study on the self-propulsion of 3D flutter flexible plates in stationary fluid by combining the immersion boundary lattice Boltzmann method and the finite element method, pointing out that proper structural flexibility can improve the propulsion speed and efficiency. Lagopoulos et al. [25] revealed that increasing the aspect ratio can improve thrust coefficients and thrust augmentation by using a three-dimensional numerical simulation method. Kumar et al. [26] used the fluid module of the ANSYS software to calculate the thrust generated by the active flapping motion in short waves. The results show that an active flapping-foil can effectively convert wave energy into short wave propulsion energy, and the thrust is related to the frequency of the flapping-foil. The above numerical simulation technology has the advantages of low cost and high visualization, but it is affected by input parameters, grid quality, model accuracy, and other aspects. Therefore, numerical simulation is generally used as an auxiliary design method in bionic propulsion research and is very suitable for performing underwater tasks that require high control accuracy and high concealment.

Experimental research is an indispensable and important technical means for bionic flapping-foil propulsion [27]. Koochesfahani [28] observed the flow field of a NACA0012 wing with sinusoidal and non-sinusoidal pitch swing in a low-speed water tunnel. It was found that the tail vortices were arranged in reverse Karman vortex street when the frequency gradually increased to a certain extent, which is manifested as thrust. Parker et al. [29] used the Particle Image Velocimetry (PIV) technology to observe the flow field and wake during the flapping of a three-dimensional symmetrical NACA wing with limited span length. Based on the experimental results, the geometrical characteristics of the flow around the wing were described and an eddy current motion model was established. Mumtaz et al. [30] investigated the fluid structure coupling (FSI) and energy extraction performance of a novel flapping wing-based flow energy harvester through experiments. Unlike most existing concepts, this device can extract energy from the flow through the completely passive flapping motion of the hydrofoil, meaning that the undulating and pitching motion of the hydrofoil is caused by the flow without the need for any actuators. Thaweewat et al. [31] used experimental methods to study a semi-active flapping wing for ocean propulsion and explored the effects of different control strategies on thrust coefficient and propulsion efficiency. Izraelevitz et al. [32] studied the effect of adding linear oscillating motion to the oscillating undulations and pitching motions of a flapping-foil. The experimental results show that this method can not only increase average lift significantly, but also reduce oscillating lift, increasing thrust and propulsion efficiency. Mackowski et al. [33] discussed the influence of elastic heave motion on the propulsion characteristics of a controllable pitching wing by placing passive springs on the wing in the heave direction, which provided a new idea for the flexible design of the wing. Zhou et al. [34] studied the influence of motion parameters such as the angle of attack and stroke angle on the hydrodynamic performance of the flapping-foil, and compared the results with those of numerical calculations, which were in good agreement.

All of the above research have promoted the exploration of the propulsion mechanism of the bionic flapping-foil. In this study, a unique design method was proposed. In order to allow only one of the thrust or lift to be measured simultaneously, the propulsion system and data acquisition system are connected through connecting plates and guide rail slider mechanisms. Only the force in the direction of the slider movement is retained, thus the thrust, lift, and torque are decoupled. When the equilibrium position of the flapping-foil cycle is parallel to the *x*-axis, the measured force is thrust. The force measured when rotating the foil by 90° so that the flapping balance position is perpendicular to the *x*-axis is called lift. However, during the force measurement process, the force perpendicular to the *x*-axis may increase the frictional force of the slider, causing minor errors in the measurement of thrust and lift. And, the effect of the frequency–amplitude parameter and aspect ratio on the propulsion performance of the underwater flapping-foil is studied to explore and reveal the bionic flapping-foil propulsion mechanism. This study can provide a theoretical reference and technical support for the design of new bionic flapping-foils.

## 2. Materials and Methods

### 2.1. Experiment Setup

**Figure 1:**
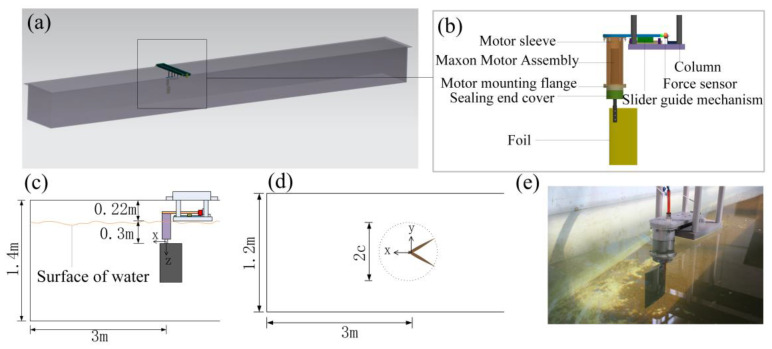
Structure and layout of hydrodynamic experimental platform: (**a**) Experimental platform model. (**b**) Experimental device structure. (**c**) Installation diagram in Y direction. (**d**) Installation diagram in Z direction. (**e**) Experimental device physical image.

As shown in Figure 1a, the flapping-foil hydrodynamic performance experimental platform designed in this study is based on the experimental water tank. The length, width, and depth of the water tank are 10 m, 1.2 m, and 1.4 m. The overall structure and connection of the experimental device are shown in Figure 1b. The device is installed at the position shown in Figure 1c–e, and the leading edge of the foil is 3 m away from the front of the tank and the tail of the foil is 7 m away from the back of the tank. The tail of the foil is provided with enough length in all directions from the water tank to ensure the full development of the wake during the flapping-foil movement. When the water tank is filled to the lower edge of the foil, the top of the foil is about 0.3 m away from the water surface, which can avoid the influence of the water surface fluctuation caused by the foil swing on the measurement results. The maximum swing range of the foil is twice that of the chord length of the foil (*c*), and the width of the tank is about 6–12 times the chord length. In addition, the force sensor is placed above the water surface to reduce unnecessary problems caused by watertight treatment.

The whole experimental device can be divided into two parts: the first is the flapping-foil propulsion and control system and the second is the data acquisition and storage system. The propulsion and control system is used to drive and control the foil to flap according to the established motion law in water and is mainly composed of the foil to be measured, drive motor components (Maxon motor, reducer, encoder, and driver), sealing device, and so on. The data acquisition and storage system is used to acquire analog signals output by sensors and convert them into digital signals for storage. It is mainly composed of a force sensor, signal amplifier, DAQ data acquisition card, and computer. The propulsion system and data acquisition system are connected by a connecting plate and a guide rail slider mechanism, which decouples thrust, lift force, and torque and only retains the force along the direction of the slider movement, which is in the direction of the *x*-axis and acts on the force sensor via the connecting plate. When the balance position of the periodic swing of the flapping-foil is parallel to the *x*-axis, the force measured is thrust. When the foil is rotated 90°, the force measured when the flapping balance position is perpendicular to the *x*-axis is the lift force.

**Figure 2:**
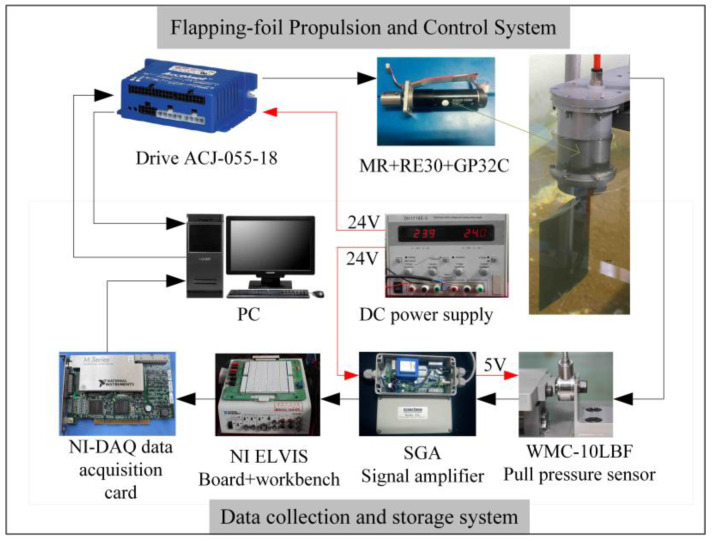
Experimental device connection diagram.

The signal transmission, power supply, and work flow of the experimental device are shown in Figure 2. An encoder (MR) (Maxon, Sachsen, Switzerland), DC servo motor RE30 (Maxon, Sachsen, Switzerland), and reducer (GP32C) (Maxon, Sachsen, Switzerland) are selected as the motor components of the flapping-foil propulsion system, and the small column high-precision tension pressure sensor WMC-10LBF (INTRERFACE, Arizona, America) is selected as the force sensor and added to the amplification circuit. Proprietary temperature-compensated strain gages are used by the WMC, enabling it to operate within the ambient temperature range of −10 °C to 45 °C. Due to the fact that the output voltage of the force sensor is measured in millivolts, an amplification circuit should be added before data acquisition to reduce noise interference caused by cables and other environmental factors during signal acquisition. This article selects a signal amplifier SGA and amplifies the numerical range of the tension and pressure voltage signal transmitted back by the WMC to ±5 volts. Before the experiment, the sensor is calibrated, and then the ratio of force to SGA output signal voltage is 1 kg/V. The SGA includes a second-order low-pass filter with a filtering range of 1 Hz~5 kHz. Reasonable setting of the filter frequency can effectively reduce the interference of electromagnetic noise and high-frequency signal fluctuations in the experimental environment on measurements. In the experiment, the maximum flapping frequency was 4 Hz, and the thrust change frequency was 8 Hz. Therefore, the filtering frequency of the filter was set to 10 Hz. The PCI-6251 data acquisition card is used as a data collector and its data acquisition and A/D conversion are controlled by the Labview software (2014 SP1) and NI-DAQmx drive (Ni, Austin, TX, USA). Motion control commands are sent to the motor drive ACJ via the PC. The values of voltage, current, position, and speed of motor operation are received by the PC. The hydrodynamic signal returned by the sensor is collected and stored by the PC through the DAQ acquisition system.

### 2.2. Data Processing Method

When measuring the hydrodynamic force of the flapping-foil, 10 s are sampled in each working condition with a sampling frequency of 1200 Hz. The voltage analogue sampled is converted to obtain the experimental data of thrust and lift under this working condition, and the curve diagram changing with time is plotted. For the sampling data which still has noise interference after hardware filtering, a spectrum analysis is carried out and software filtering is carried out according to the results of the spectrum analysis.

**Figure 3:**
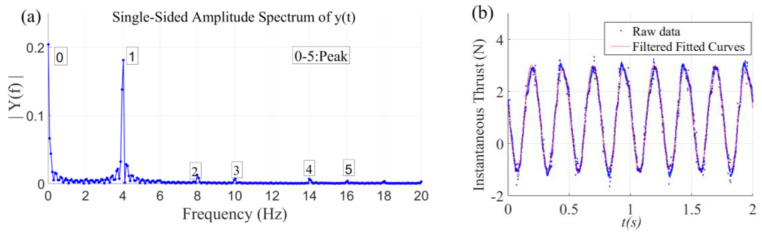
Signal spectrum analysis diagram and thrust variation curve: (**a**) Signal spectrum analysis diagram. (**b**) Thrust variation curve.

Taking a section of a NACA0012 airfoil whose chord length and extension are 100 mm and 200 mm as an example, Figure 3a shows the amplitude spectrum of frequencies obtained by fast Fourier transform based on time–frequency data when the swing frequency is 2 Hz and amplitude is 30°, where the amplitude spectrum reflects the single peak amplitude of each harmonic component in the frequency amplitude. It can be seen that the amplitudes of point 0 and point 1 are the largest and the corresponding frequencies are 0 Hz and 4 Hz, respectively, which indicate that the signals of frequency 0 Hz and 4 Hz play a leading role in the data acquisition. Figure 3b shows the instantaneous change curve of thrust with time when the swing frequency of the flapping-foil is 2 Hz and the amplitude is 30°. It can be seen that the noise points of the thrust fitting curve after filtering are basically removed, and the hydrodynamic signal is well restored. The thrust pulsation frequency is 4 Hz, twice that of the flapping-foil. The average thrust is positive, indicating that the flapping-foil swing produces a net thrust, which is consistent with the spectrum analysis results above.

## 3. Results and Discussion

### 3.1. Effects of Frequency and Amplitude

In this section, a NACA0012 flapping-foil with a chord length of 100 mm and flapping-foil plate span of 200 mm was used as the experimental object to study the effect of frequency and amplitude on the hydrodynamic performance. There are four values of the frequency, 1 Hz, 2 Hz, 3 Hz, and 4 Hz. The amplitude range is 5°~60°, and every 5° is a measurement point. So, there are 48 kinds of working conditions. In the experiment, it is found that when the flapping frequency is 4 Hz and the amplitude is greater than 40°, the amplitude tracking of the driving motor is distorted, and the instantaneous peak value of the hydrodynamic force slightly exceeds the sensor range. So, the four working conditions of 4 Hz-45°, 4 Hz-50°, 4 Hz-55°, and 4 Hz-60° are removed when processing the results.

**Figure 4:**
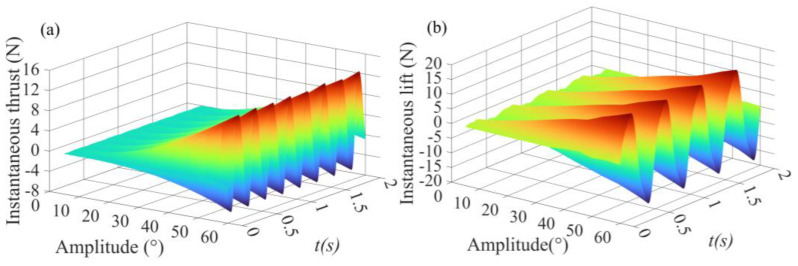
Instantaneous hydrodynamic force at 2 Hz: (**a**) Instantaneous thrust. (**b**) Instantaneous lift.

The instantaneous hydrodynamic simulation signal values measured under the 44 working conditions and the instantaneous output voltage, current, speed, and position of the driving motor under each working condition are recorded and saved by the measurement system. Every working condition is repeated twice and processed following the method of Section 2.2. Referring to the coordinate system shown in Figure 1c,d, the force along the *x*-axis direction generated by the flapping-foil swing pushes the vehicle forward, which is defined as thrust; the hydrodynamic force generated along the *y*-axis direction causes the vehicle to move up and down, defined as lift. Figure 4a,b shows the instantaneous thrust and instantaneous lift change with time when the flapping-foil swing frequency is 2 Hz.

**Figure 5:**
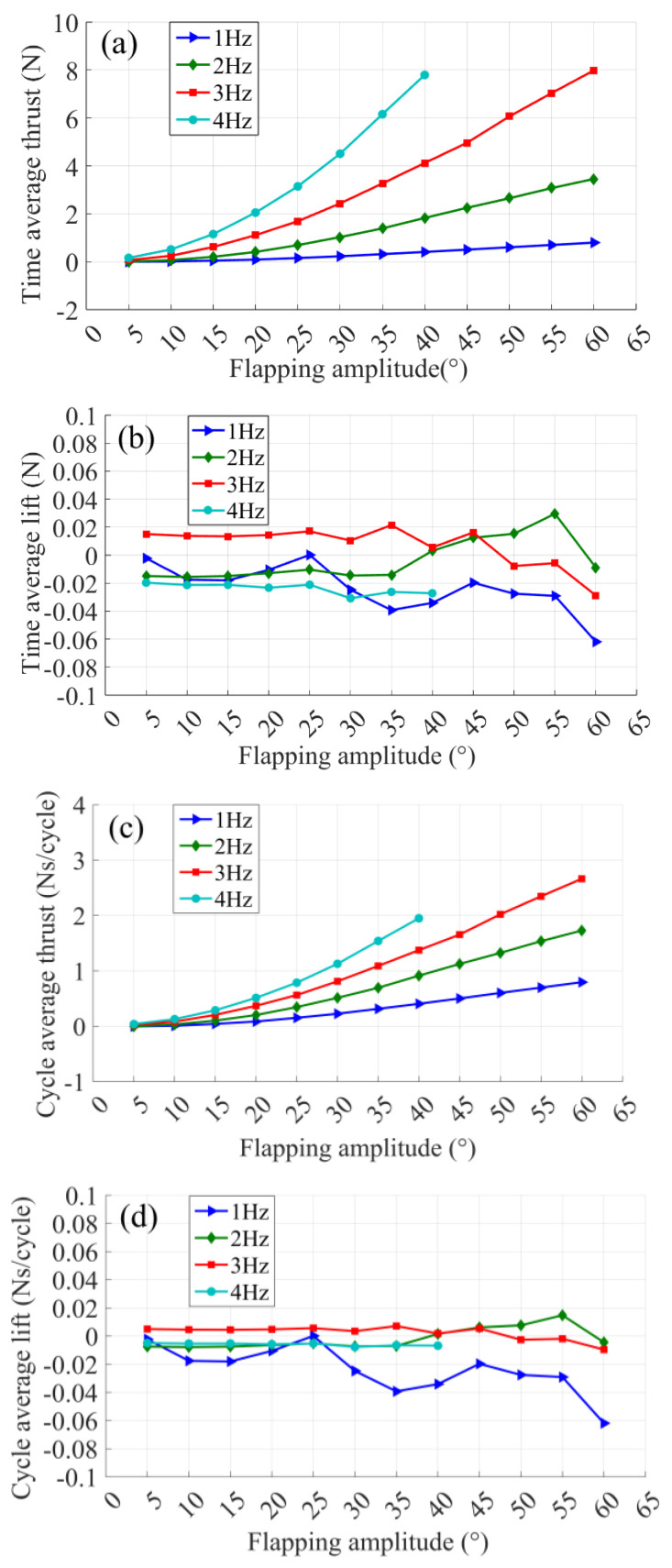
Experimental results of time and cycle average thrust (lift): (**a**) Time average thrust. (**b**) Time average lift. (**c**) Cycle average thrust. (**d**) Cycle average lift.

Then, the integral of instantaneous thrust (lift) force obtained in an integer cycle is divided by the time taken to obtain the time-averaged thrust (lift) force, which represents the net thrust (lift) force generated by the flapping-foil movement in a unit time, as shown in Figure 5a,b. The average time thrust (lift) force is divided by the number of flapping-foil oscillation cycles per unit time to obtain the flapping-foil periodic thrust (lift) force, which represents the average thrust (lift) force generated by one flapping oscillation cycle, as shown in Figure 5c,d. From Figure 5a, it can be seen that the time-averaged thrust increases with the increase of the flapping frequency and amplitude. From Figure 5b, it can be seen that the time-averaged lift varies independently of the frequency and amplitude of the flapping-foil oscillation and approaches zero under all experimental conditions. At the same time, the greater the flapping frequency, the more cycles the flapping-foil will go through. If the net thrust generated by the two flapping motions with the amplitude is equal, it is obvious that the higher the flapping frequency, the greater the average thrust generated per unit time of the flapping-foil. However, as can be seen from Figure 5c, the shorter the flapping duration, the greater the average thrust generated in a single cycle of the flapping-foil.

With the same two oscillations, the larger the flapping amplitude, the larger the average thrust generated in a single cycle, resulting in the increase of the average thrust of flapping time with the increase of flapping frequency and amplitude, and the growth rate of the average thrust increases gradually, which leads to the nonlinear increase of the average thrust with the increase of the flapping frequency and amplitude. Under all experimental conditions, the average lift force tends to zero in a single cycle. From Figure 5d, it can be seen that no net lift force is generated in a single cycle when a symmetrical foil moves in a symmetrical motion.

**Figure 6:**
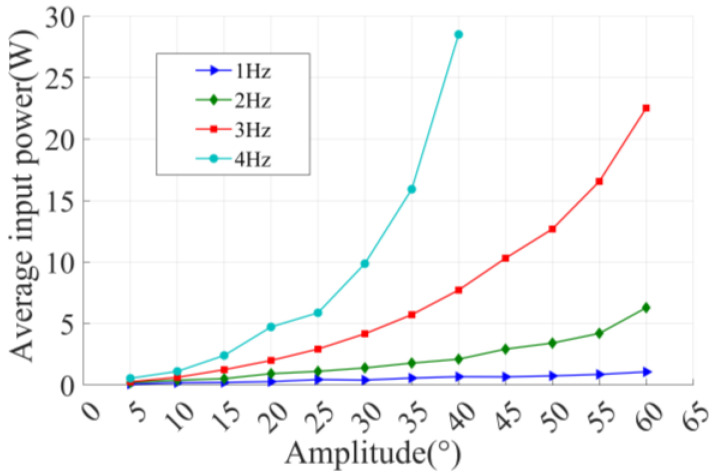
Diagram of the relationship between average input power and motion parameters.

The power required for the flapping-foil is input by the servo motor. In the experiment, the actual current and rotating speed of the servo motor are recorded by the software to calculate the input power of the foil. The torque constant value of the Maxon RE30 motor (Maxon, Sachsen, Switzerland) used in this experiment is 25.9 mNm/A, and its instantaneous torque *T* is the product of the torque constant and current. The relationship between the power consumed per unit time of flutter and the motion parameters obtained by processing the experimental data is shown in Figure 6.

A flapping-foil can generate more net thrust per unit time with a large amplitude and high frequency, but the power required to drive a flapping-foil at high frequencies or large amplitudes is also greater. Therefore, it is necessary to measure the propulsive efficiency of the flapping-foil, so as to make it clear in what motion state the flapping-foil can generate more thrust with the minimum input power. The definition of propulsion efficiency is expressed by the following formula:

(1) $$\eta =\frac{{\bar{C}}_{T}}{{\bar{C}}_{P}}$$

where ${\bar{C}}_{T}$ and ${\bar{C}}_{P}$ are the average thrust coefficient and the average input power coefficient for a single cycle, respectively, which are represented by the following formulas:

(2) $${\bar{C}}_{T}=\frac{1}{T}\int_{0}^{T}C_{T}(t)dt=\frac{1}{T}\int_{0}^{T}\frac{F_{x}(t)}{0.5\rho U^{2}cb}dt$$

(3) $${\bar{C}}_{P}=\frac{\bar{P}}{0.5\rho U^{3}cb}$$

where *ρ* is the density of water, *U* is the input flow speed, *c* is the chord length of the foil, *b* is the foil span length, and *F_x_*(*t*) is the component of the thrust in the *x*-axis. The input power of the foil is obtained by converting the actual current and speed values of the servo drive motor recorded by the computer software.

Further, combining Formulas (1)–(3) yields:

(4) $$\eta =\frac{{\bar{C}}_{T}}{{\bar{C}}_{P}}=\frac{{\bar{F}}_{x}\cdot U}{\bar{P}}$$

Average thrust ${\bar{F}}_{x}$ and average input power $\bar{P}$ can be obtained directly by experiments, while *U* is taken as the relative incoming flow velocity in the experiment. Since all the flapping-foil hydrodynamic performance tests in this paper are carried out in hydrostatic water, a dimensionless treatment is carried out on *U* as unit “1”. The calculated propelling efficiency is not the usual propelling efficiency. It is specially recorded as *η_still_*:

(5) $$\eta_{still}=\frac{{\bar{F}}_{x}}{\bar{P}}$$

**Figure 7:**
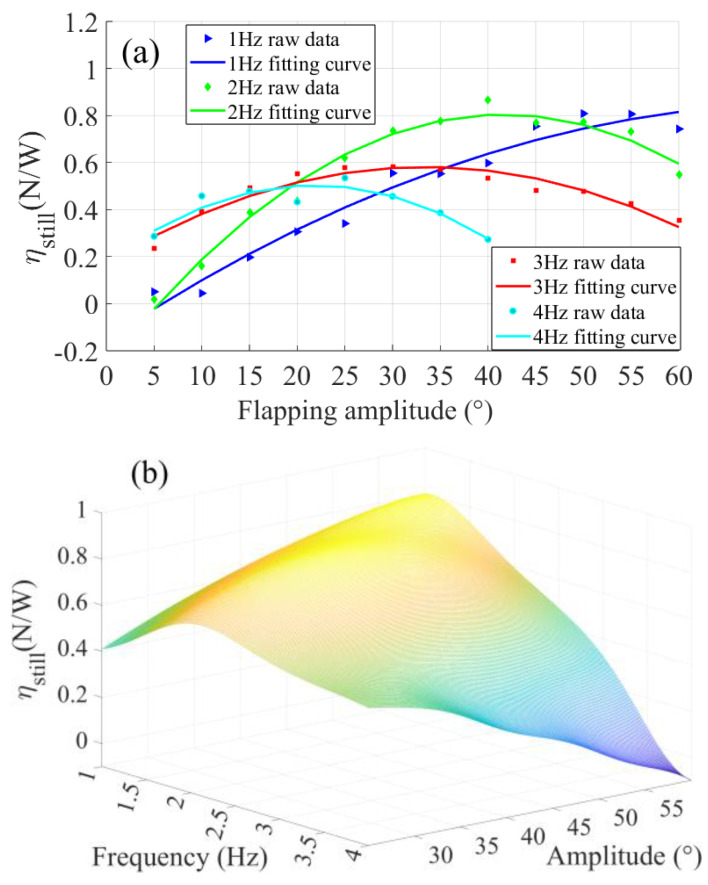
Flapping-foil propulsion efficiency with different frequency–amplitude motion parameters: (**a**) Experimental results and fitting curves of propulsion efficiency. (**b**) Response surface of propulsion efficiency to motion parameters.

*η_still_* is a dimensioned variable and its unit is N/W. Its physical meaning is the thrust generated by unit input power, which can well reflect the flapping-foil propulsion performance from the perspective of energy conversion. Figure 7 shows the relationship between the experimental flapping-foil propulsion efficiency and motion parameters and divides all 44 operating conditions into four groups according to the flapping frequency. As can be seen from Figure 7a, when the flapping frequency is 1 Hz, the flapping efficiency increases with the increase of flapping amplitude and the increase rate decreases gradually. However, the highest efficiency point appears in all the other three groups of operating conditions. Compared with the position where the highest efficiency point appears, when swing frequency is 2 Hz, the corresponding flapping amplitude of the highest efficiency point is about 40°. When the flapping frequency is 3 Hz, the corresponding flapping amplitude is about 30°. When the flapping frequency is 4 Hz, the corresponding flapping amplitude is about 20°. The relationship between propulsion efficiency and motion parameters is represented by the response surface shown in Figure 7b. Under all experimental conditions, the maximum flapping propulsion efficiency is 0.87 N/W at a frequency of 2 Hz and an amplitude of 40°.

The Strauhal number for flapping movement is defined as follows:

(6) St=fA/U=2fcsinθ/U

where *f* is the frequency, *A* is the wake width, and *θ* is the amplitude. Similarly, *U* is dimensionless as unit “1”. According to the results and Formula (6) in Figure 7, the *St* values corresponding to the maximum propulsion efficiency at 2 Hz, 3 Hz, and 4 Hz are calculated as 0.257, 0.3, and 0.274, respectively. This is consistent with the conclusion in the literature that the maximum flapping-foil propulsion efficiency appears when the *St* values are within the range of 0.2–0.4 [35].

### 3.2. Effects of Aspect Ratio

**Figure 8:**
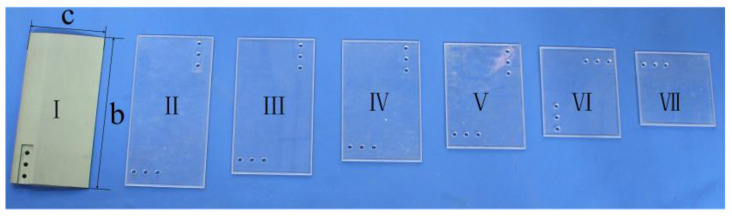
Foil models with different aspect ratios.

In order to investigate the influence of foil size on the hydrodynamic performance of the flapping-foil, hydrodynamic performance tests were carried out for different aspect ratios (*c*/*b*) in this study. The foil models are shown in Figure 8. The foil I section shape is NACA0012, made of an aluminum alloy with 100 mm chord length and 200 mm span length. The foils II~VII are thin rectangular foils made of organic glass and the specifications of the span lengths (*c*) and chord lengths (*b*) are 200 × 100 mm, 180 × 100 mm, 160 × 100 mm, 140 × 100 mm, 120 × 100 mm, and 100 × 100 mm, respectively.

**Figure 9:**
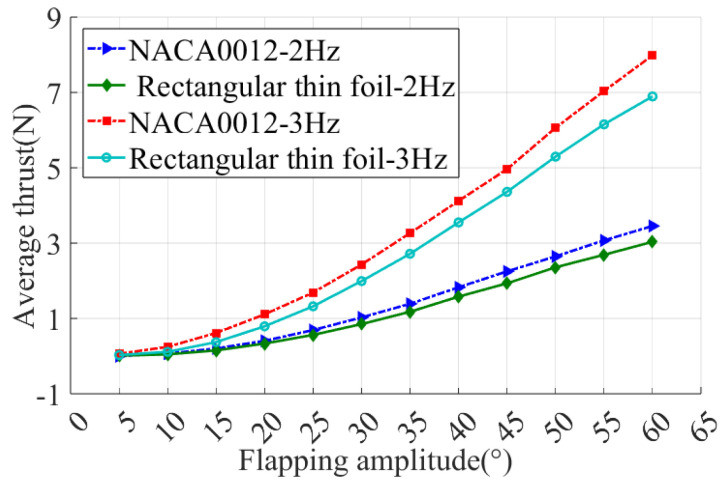
Propulsion performance comparison of NACA0012 and rectangular thin foil.

The difference between foils can mainly be summarized by their cross-sectional shape and aspect ratio. In order to verify the validity of the rectangular thin foils, experiments were carried out to compare the hydrodynamic performance of foil I and foil II, and the curves of the average thrust of the two foils varying with the motion parameters were measured as shown in Figure 9. It can be seen from the figure that the propulsion performance of the rectangular thin foil is slightly lower than that of the NACA0012 foil, but the overall difference is small. Therefore, for the sake of simplicity, the rectangular thin foil (II~VII) is subsequently used to study the propulsion performance with different aspect ratios.

In order to compare the influence of foil size changes in the span length and chord length directions on the hydrodynamic performance of the flapping-foil, a group of mounting holes are machined in both the length and width directions of each group of foils, and the distance between the mounting holes and the flapping leading edge is equal, as shown in Figure 8. During the experimental installation, if the span length is greater than the chord length, it is called “forward installation” flapping. If the chord length is greater than the span length, it is called “reverse installation” flapping. When the six groups of foils are “forward installation” flapping, the chord length remains unchanged at 100 mm, and the span length changes from 100 mm to 200 mm with an interval of 20 mm. When the six groups of foils are “reverse installation” flapping, the span length remains unchanged at 100 mm, and the chord length changes from 100 mm to 200 mm with an interval of 20 mm.

**Figure 10:**
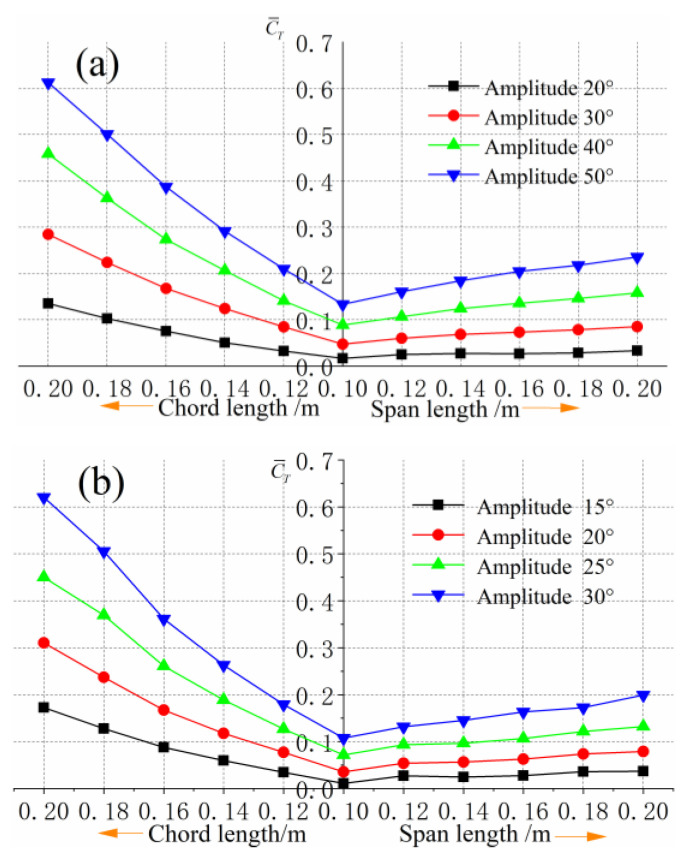
Comparison of average thrust coefficients of different sizes of foils: (**a**) Average thrust coefficient at 2 Hz. (**b**) Average thrust coefficient at 3 Hz.

As can be seen from Figure 10, when the span length is fixed, the average thrust coefficient increases with the increase of chord length, and the growth rate tends to increase. When the chord length is fixed, the average thrust coefficient slightly increases with the increase of the span length, while the growth rate gradually decreases. The reason for this change rule is that when the chord length of the foil is increased by a certain length, it is equivalent to adding an additional area in the chord direction of the foil. Under the same motion parameters, the flapping amplitude and flapping linear velocity of this additional foil increases with the increase of the chord length, and the additional thrust generated also increases, resulting in the rapid increase of the average thrust coefficient of the entire foil with the increase of the chord length. When the chord length is fixed and the span length increases, an additional area of the foil is added to the foil span direction. However, the increase of the span length will not cause the change of the motion state of this part of the additional foil, and the flow state of each section of the whole foil perpendicular to the span direction is similar, ignoring the interference of the flow around the end. The two-dimensional flapping-foil can be seen as the situation where the aspect ratio AR tends to infinity. With the infinite increase of the span length, the average thrust coefficient of the three-dimensional flapping-foil is closer to the two-dimensional situation. This is the reason that the average thrust coefficient increases slightly and gradually tends to a fixed value when the chord length is fixed and the span length is continuously increased.

For the same foil, “reverse installation” flapping can produce more thrust than “forward installation” flapping. However, when the foil is “reverse installation” flapping, the pressure center moves backward. The greater the chord length, the closer the pressure center is to the rear edge of the flapping-foil, and the greater the torque required to drive the flapping-foil, which will inevitably lead to an increase in input power. In addition, when the chord length or span length is changed, additional input power is required to drive the additional foil, and the total input power is also increased. Therefore, it is necessary to compare and analyze the propulsion efficiency of different foils.

**Figure 11:**
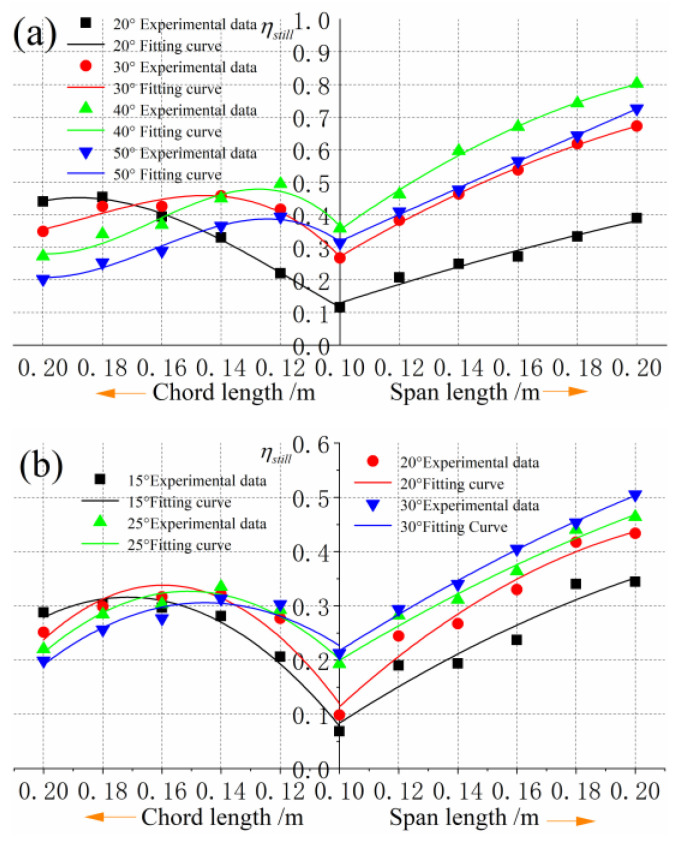
Comparison of propulsion efficiency of different sizes of foils: (**a**) Propulsion efficiency at 2 Hz. (**b**) Propulsion efficiency at 3 Hz.

The experimental results of propulsion efficiency are shown in Figure 11. When the chord length is fixed and the span length increases, the propulsion efficiency increases gradually, but the growth rate decreases gradually. According to the previous analysis, when the span length is infinite and the average thrust coefficient tends to be constant, then the average thrust is proportional to the span length. However, when the flow state of each section in the direction of span is similar, then the input power is proportional to the span length. Therefore, it can be inferred that when the span length continues to increase, the growth trend of propulsion efficiency becomes slower and tends to be fixed gradually. With a certain extension, the propulsive efficiency increases first and then decreases with the increase of chord length, and the highest efficiency appears. At the same flapping frequency, the position of the highest point of propulsion efficiency shifts to the right with the increase of flapping amplitude. The larger the flapping amplitude, the smaller the chord length corresponding to the highest point of propulsion efficiency and the larger the aspect ratio of the aspect ratio.

## 4. Conclusions

Bionic flapping propulsion offers a novel propulsion method for UUVs. This research introduces the development of a hydrodynamic experimental platform that simulates the motion of a flapping-foil, which consists of a propulsion mechanism and a data acquisition setup. They are connected through a connecting plate and a guide rail slider mechanism. This guide rail slider mechanism can decouple thrust, lift, and torque, retaining only the force along the direction of the slider’s movement. Then, the effect of the frequency–amplitude parameter and aspect ratio on propulsion performance of the underwater flapping-foil is analyzed.

The results indicate a nonlinear increase in the thrust generated by the flapping motion, which correlates with an escalating rate of increase as frequency and amplitude rise. Among the 44 tested operating conditions, the maximum thrust coefficient of 0.87 N/W was achieved using a NACA0012 foil at a frequency of 2 Hz and an amplitude of 40 degrees. Additionally, it was discovered that the “reverse installation” configuration of the foil, where the foil flaps in the opposite direction of its intended design, yields a higher thrust compared to the “forward installation” configuration. However, this reverse configuration also causes the pressure center to shift backwards. Furthermore, when the span length remains unchanged, an increase in chord length leads to a higher average thrust coefficient, with the propulsive efficiency peaking at a certain chord length. Conversely, when the chord length is held constant, both the average thrust coefficient and propulsive efficiency improve with an increase in span length, albeit with a diminishing rate of growth. The study underscores the significant potential of bionic flapping-foil technology for advancements in the field of underwater propulsion, highlighting its importance for future research and applications.

## Data Availability

Raw and derived data supporting the findings of this study are available from the corresponding author upon reasonable request.
